# Efficacy of Electronic Acupuncture Shoes for Chronic Low Back Pain: Double-Blinded Randomized Controlled Trial

**DOI:** 10.2196/22324

**Published:** 2020-10-26

**Authors:** Bo-Yan Yeh, Geng-Hao Liu, Tzung-Yan Lee, Alice May-Kuen Wong, Hen-Hong Chang, Yu-Sheng Chen

**Affiliations:** 1 Division of Acupuncture and Moxibustion, Department of Traditional Chinese Medicine Chang Gung Memorial Hospital, Linkou Taoyuan Taiwan; 2 School of Traditional Chinese Medicine Chang Gung University Taoyuan Taiwan; 3 Sleep Center Chang Gung Memorial Hospital Taoyuan Taiwan; 4 Graduate Institute of Traditional Chinese Medicine, School of Chinese Medicine, College of Medicine Chang Gung University Taoyuan Taiwan; 5 Department of Traditional Chinese Medicine Chang Gung Memorial Hospital Keelung Taiwan; 6 Department of Physical Medicine and Rehabilitation Chang Gung Memorial Hospital Taoyuan Taiwan; 7 Graduate Institute of Integrated Medicine, College of Chinese Medicine, and Chinese Medicine Research Center China Medical University Taichung Taiwan; 8 Department of Chinese Medicine China Medical University Hospital Taichung Taiwan; 9 Graduate Institute of Clinical Medical Sciences Chang Gung University Taoyuan Taiwan

**Keywords:** acupuncture, electronic acupuncture shoes, low back pain, medical device, self-treatment, mHealth

## Abstract

**Background:**

Chronic low back pain is a common problem and is associated with high costs, including those related to health care and indirect costs due to absence at work or reduced productivity. Previous studies have demonstrated that acupuncture or electroacupuncture can relieve low back pain. Electronic acupuncture shoes (EAS) are a novel device designed in this study. This device combines the properties of acupuncture and transcutaneous electrical nerve stimulation for clinical use.

**Objective:**

The aim of this study was to evaluate the efficacy of EAS in patients with chronic low back pain.

**Methods:**

In this prospective double-blinded randomized controlled study, the data of 83 patients who experienced chronic low back pain were analyzed. Patients came to our clinic for 20 visits and underwent assessment and treatment. Patients were randomly allocated to receive either EAS plus placebo nonsteroidal anti-inflammatory drugs (NSAIDs) (EAS group, n=42) or sham EAS plus NSAIDs (NSAID group, n=41). The visual analog scale (VAS) score and range of motion were assessed at baseline, before and after each EAS treatment, and 2 weeks after the last treatment. The time for achieving pain remission was recorded. Quality of life was assessed at the 2nd, 14th, and 20th visits.

**Results:**

After 6 weeks of treatment, the treatment success rate in each visit in the EAS group was higher than that in the NSAID group, as revealed by the intention-to-treat (ITT) and per-protocol (PP) analyses, but significant differences were observed only during the 16th visit in the ITT analysis (EAS group: 31/37, 84% and NSAID group: 21/34, 62%; *P*=.04). The change in the VAS score from baseline in each visit in the EAS group was greater than that in the NSAID group, as revealed by the ITT and PP analyses, and significant differences were observed in the 5th visit and 9th visit in the ITT analysis (*P*=.048 and *P*=.048, respectively). Significant differences were observed in the left rotation in the 2nd visit and 4th visit (*P*=.049 and *P*=.03, respectively). No significant differences were observed in the VAS score before and after treatment in each visit and in the quality of life in both groups.

**Conclusions:**

EAS might serve as a reliable alternative therapeutic tool for patients with chronic low back pain who are contraindicated for oral NSAIDs.

**Trial Registration:**

ClinicalTrials.gov NCT02468297 https://clinicaltrials.gov/ct2/show/NCT02468297

## Introduction

Low back pain (LBP) is a common problem worldwide, and its prevalence ranges from 22% to 48% [1]. The lifetime prevalence of LBP is 84% [2]. LBP is associated with high costs, including those related to health care and indirect costs from missed work or reduced productivity [1]. Acupuncture is a cost-effective treatment strategy for chronic LBP (CLBP) [3]. CLBP is commonly defined as back pain that persists for at least 12 weeks. In clinics, physicians usually prescribe nonsteroidal anti-inflammatory drugs (NSAIDs) to relieve pain. However, these NSAIDs cause side effects such as nausea, peptic ulcer, gastrointestinal bleeding, and elevated blood pressure.

Lee et al [4] proposed that acupuncture is a simple and effective strategy for relieving pain, but it cannot improve the loss of function and disability resulting from LBP. The guidelines of the American College of Physicians strongly recommend acupuncture for the selective nonpharmacologic treatment of CLBP [1]. The mechanism of acupuncture analgesia is associated with central neurotransmitters, immune cytokines, and cytokines from the spinal glial cells [5]. These substances can produce various effects such as analgesic, muscle relaxation, anti-inflammatory, mild anxiolytic, and antidepressant effects [6].

Electroacupuncture is defined as applying electrical stimulation to acupuncture needles [6]. This strategy may result in a faster analgesic and anesthetic effect, and high-frequency electroacupuncture has been reported to control pain more effectively than low-frequency electroacupuncture [7]. Electronic acupuncture shoes (EAS) are a newly designed device; these shoes show a combination of the properties of electroacupuncture and transcutaneous electrical nerve stimulation (TENS) and employ a pain-controlling mechanism different from that of acupoint TENS [8-10].

Electronic systems have been incorporated as a component in many medical devices. For example, the electronic system in the smart assistive knee brace serves as a driving force through heating and cooling on the shape memory alloy to induce a cycle of contraction and elongation [11]. However, in the EAS, the electronic system plays the key role in the therapeutic effect by applying the current loop between KI1 and *shimian* to induce reactions in the human body through the central [12,13] and peripheral pathways [14-16], respectively. The electronic system is incorporated into the shoes with an appropriate parameter setting such that the location of the electrical stimulation is accurate. If a person contracts a new type of infectious disease such as COVID-19, EAS can serve as a very good modality for home health care and can provide therapeutic effects immediately with few contraindications. This device can be used for patients who fear acupuncture needles, and it can be manipulated by patients themselves. Our study compared the noninferiority effects of EAS with NSAIDs for the treatment of patients with CLBP.

## Methods

### Patient Selection

This study was approved by the Institutional Review Board of the Chang Gung Medical Foundation, and this trial was registered at the ClinicalTrials.gov website (NCT02468297). We registered this trial retrospectively because of the following reasons. We began to enroll patients in April 2004 but stopped in November 2008. We needed to ensure that the amplitude of the electric current was around an appropriate range. Thus, the trial protocol was revised for performing a test to determine the resistance of the sole of every participant. After the revision of the protocol, we re-enrolled patients in April 2009. The manufacturer of EAS did not want to disclose the trade secret before the trial had been completed.

Informed consent was obtained from each participant. A total of 90 patients of both sexes were prospectively screened for study participation. All patients were outpatients of the Department of Rehabilitation, Orthopedics, and Chinese Medicine. We selected patients with the following inclusion criteria: diagnosis of CLBP, that is, the location of pain was below the 12th rib and above the horizontal gluteal crease and lasted for more than 12 weeks; age range of 20 to 60 years; and provision of signed informed consent. Patients were excluded if he or she met the following medical conditions, including cancer, rheumatoid arthritis, renal stone, diabetes mellitus, pacemaker implantation, under steroid treatment, fracture or surgical history of the back, spinal cord compression syndrome (eg, herniated intervertebral disc or spinal cord disorder), visceral organ infectious disease (eg, pancreatitis and pyelonephritis), or visual coordination disorder. Women who experienced menopause before 50 years of age, underwent ovary excision, or were pregnant were excluded. Further, women considering to be pregnant were asked to not enroll, and enrolled women were asked to agree to contraception or abstinence. Moreover, before joining the trial, their pregnancy test must be negative. Patients were also excluded if they were not free from previous participation in other trials within 30 days before joining this trial, showed contraindications to ibuprofen, or if they had poor heart, liver, gastrointestinal tract, and renal function. Further, if the physician suspected that EAS treatment might have adverse effects on the patient based on the physical examination and laboratory data at the first visit, the patient was not enrolled.

### Study Setting

All patients were randomly assigned to either EAS group (EAS plus placebo NSAIDs) or NSAID group (sham EAS plus NSAIDs) based on a concealed allocation approach. A computerized random number table was used to determine group allocation. Numbered opaque sealed envelopes contained ibuprofen or placebo analgesics and numbered EAS, and no restrictions were placed on randomization. The envelopes for the analgesics and EAS were kept by a pharmacist who was not an assessor of the study. All assessors and patients were blinded to group allocation.

### EAS and NSAID settings

In the first visit (first week), patients were assessed by physical examination, vital sign evaluation, blood test (complete blood count, clinical chemistry, and pregnancy test), radiography of the lumbar spine in the anterior-posterior and lateral views, deep tendon reflex test, and sensation, range of motion, and pain scales. Subsequently, patients received EAS treatment (EAS group) or placebo EAS (NSAID group) for 1 hour 3 times a week for 6 weeks. Patients were assessed at baseline (first visit), before and after each EAS treatment (2nd-19th visit), and 2 weeks after the last treatment (20th visit).

### Experimental Group

In the first week of treatment, patients in the EAS group received placebo analgesics. Before EAS treatment, patients were asked to remove any conducting metal accessories, watch, mobile, and socks. Each patient had their own EAS.

During treatment, if the patient experienced any discomfort, indicated by symptoms such as extreme LBP, spasm in both lower limbs, paralysis, tachycardia, or dizziness, the assessors recorded the symptoms and their duration. If the symptoms were slight, EAS treatment was ceased for 5-10 minutes and then continued after the symptoms were relieved. If the symptoms were severe, EAS treatment was stopped at once, and the assessors recorded the reasons for stopping the management and the treatment duration. After each treatment, the patient rested for 3-5 minutes, relaxed the joints of the lower limbs, and then underwent the efficacy assessment.

### Control Group

In the first week of treatment, patients in the NSAID group received 400 mg ibuprofen 3 times a day. Subsequently, they received EAS treatment without electric current 3 times a week for 6 weeks, as in the EAS group.

### Outcome Measurements and Follow-Up

#### Primary Outcome Measurement: Pain Intensity

Pain intensity was assessed using the visual analog scale (VAS). The VAS is an 11-point scale ranging from 0 to 10. A VAS score of 0 implies absence of pain, and a score of 10 implies unbearable pain. Patients rated their pain levels before and after each EAS treatment (2nd-19th visit); otherwise, pain levels were assessed at the first visit and at 2 weeks after the last treatment (20th visit) [17].

#### Secondary Outcome Measurements

##### Time for Achieving Pain Remission

This measurement represented the course of pain remission and was defined as the time point when the VAS score began to decrease.

##### Range of Motion

**Figure 1:**
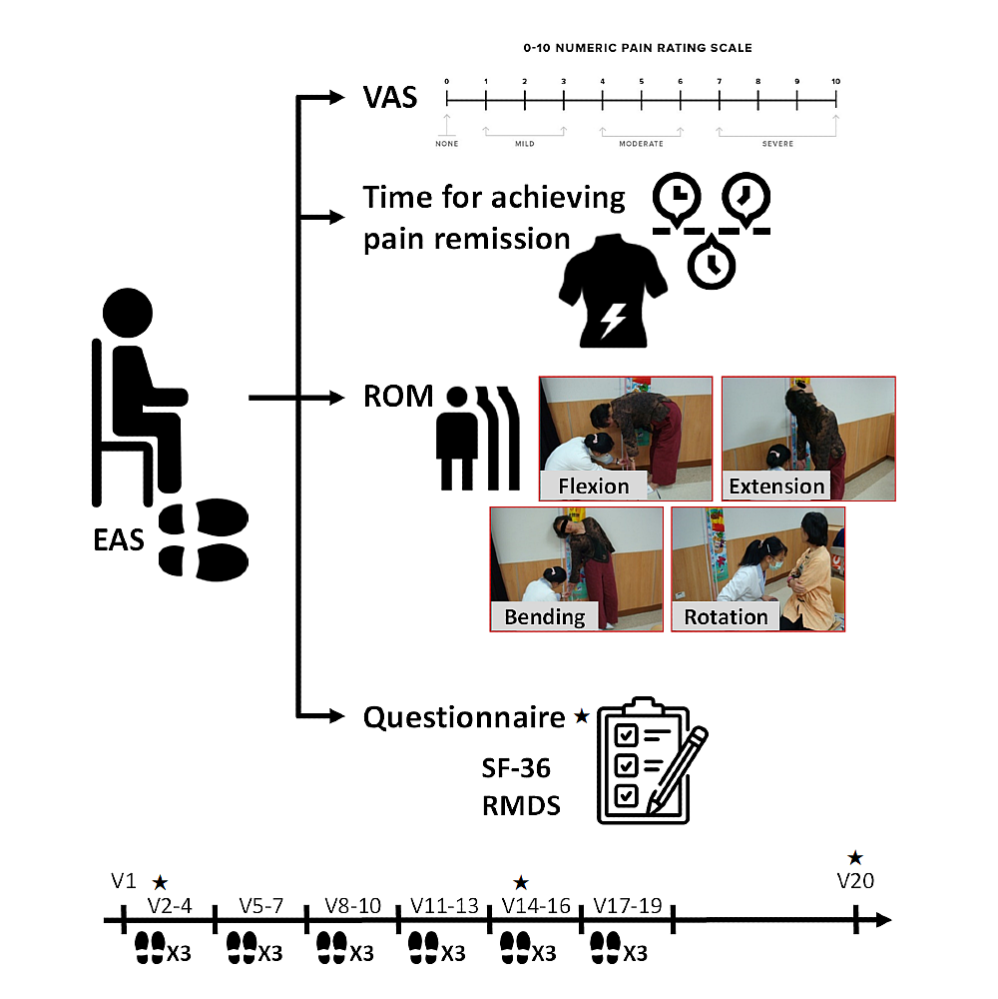
The study protocol, the circumstance of the experimental investigation, and the study assessments. Stars in the figure indicate that questionnaires were administered on the 2nd, 14th, and 20th visits. EAS: electronic acupuncture shoes; VAS: visual analog scale; ROM: range of motion; SF-36: 36-item short form; RMDS: Roland Morris Disability Scale; V: visiting day.

The patient stood upright without shoes and with both heels close together and toes slightly apart by 15°. During measurement, the knee was extended and hands were relaxed and dropped naturally. The distance from the middle finger of both hands to the floor was measured under lumbar flexion, extension, and lateral bending. Patients sat on chairs with their feet at shoulder width. Their elbows were in flexion position, and their hands were placed in front of the chest. The angle of the protractor on the wrist was measured under lumbar rotation. The range of motion of the lumbar spine was assessed before and after each EAS treatment (2nd-19th visit); otherwise, the range of motion was assessed at the first visit and at 2 weeks after the last treatment (20th visit). The study protocol, the circumstance of the experimental investigation, and the study assessments are shown in Figure 1.

##### Quality of Life

The 36-item short form (SF-36) health survey was used to assess the health-related quality of life. It consists of 36 questions grouped into 8 domains: general health (6 items), vitality (4 items), physical function (10 items), bodily pain (2 items), physical role limitation (4 items), emotional role limitation (3 items), social function (2 items), and mental health (5 items). For each domain, scores range from 0 to 100, and higher scores reflect better quality of life [18].

##### Maintaining Treatment Effect

The Roland Morris Disability Scale (RMDS) questionnaire was used to assess the functional disability due to LBP. This questionnaire consists of 24 questions that focus on the regular activities of daily living. Each affirmative answer corresponds to 1 point, and the total number of points determines the final score. The total score ranges from 0 to 24, and higher scores reflect increased disability. Scores higher than 14 indicate severe impairment [19]. The SF-36 and the RMDS questionnaire were administered to patients at the 2nd, 14th, and 20th visits.

#### Statistical Analysis

The objective of the statistical analysis was to effectively determine whether the difference between the treatment success rate of the experimental group was at least 30% more than that of the control group, where treatment success denoted VAS score after treatment being lower than that before treatment. *α* and *β* were set as .10 and .20, respectively. At a test power of 80%, the estimated effective sample size was 66. Thus, according to an experimental group-control group ratio of 1:1, the number of patients assigned to each group was 33. To reasonably assess the treatment outcome, the patients were required to undergo more than 12 treatments before their treatment outcome was included in the assessment. The SAS program (version 9.3) was used to analyze the data (SAS Institute Inc). Chi-squared test and Fisher exact test were used for the comparison of the categorical variables between the groups. For continuous variables, Student *t* test (two-tailed) was applied for 2 independent samples between the groups, while paired *t* test was chosen for within-group evaluation. The results are reported as mean (SD). A *P* value of <.05 was considered statistically significant.

## Results

### Analytic Paradigms

The population evaluated in the intention-to-treat (ITT) analysis included those who met the inclusion and exclusion criteria and who received treatment at least once without violating the protocol. The population evaluated in the per-protocol (PP) analysis was included as those receiving treatment at least 12 times without violating the protocol.

### Subject Characteristics

**Figure 2:**
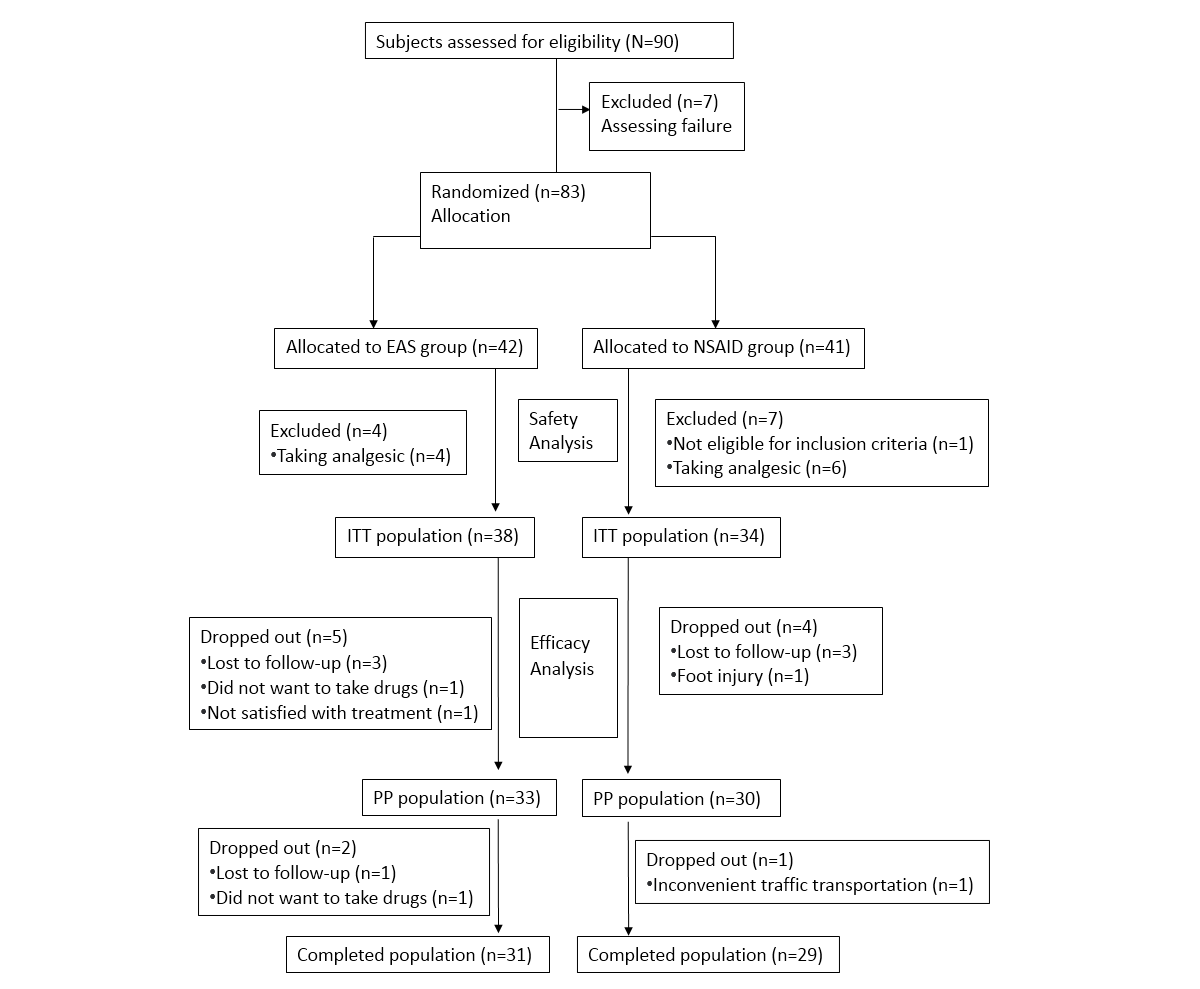
CONSORT flow diagram. CONSORT: Consolidated Standards of Reporting Trials; EAS: electronic acupuncture shoes; NSAID: nonsteroidal anti-inflammatory drug; ITT: intention-to-treat; PP: per-protocol.

Eighty-three patients were enrolled in this study between April 2009 and January 2012. One patient was excluded because of not meeting the inclusion criteria, and 10 patients were excluded because they received analgesics. The CONSORT (Consolidated Standards of Reporting Trials) (Multimedia Appendix 1) flow diagram for the study is shown in Figure 2. Patient characteristics, including age, sex, weight, height, and duration of pain, were similar between the groups, except for age in the ITT analysis, which differed significantly (EAS group, 45.7 years and NSAID group, 41.1 years, *P*=.04) (Table 1).

**Table 1:** Characteristics of the patients and duration of pain.

Characteristics		Intention-to-treat population			Per-protocol population		
		Group EAS^a^	Group NSAID^b^	*P* value	Group EAS	Group NSAID	*P* value
Patients, n		38	34		33	30	
Age (years), mean (SD)		45.7 (10.19)	41.1 (8.10)	.04^c^	45.3 (10.58)	42.0 (8.15)	.17
**Gender**				.11			.18
	Females, n	28	19		25	18	
	Males, n	10	15		8	12	
Weight (kg), mean (SD)		58.5 (11.25)	63.5 (12.52)	.08	57.4 (9.02)	62.5 (12.49)	.07
Height (cm), mean (SD)		161.8 (8.18)	164.1 (9.32)	.28	162.0 (8.25)	163.2 (9.55)	.59
Duration of pain (years), mean (SD)		8.6 (7.79)	8.5 (6.73)	.94	8.7 (8.09)	8.9 (6.75)	.94

^a^EAS: electronic acupuncture shoes.

^b^NSAID: nonsteroidal anti-inflammatory drug.

^c^Only this value was significant at *P*<.05.

### Adverse Effects

No severe adverse effects were reported in this study. Moderate and mild adverse effects such as pain in the extremities, back pain, hypoesthesia, and arthralgia were recorded. As shown in Table 2, no significant differences were observed between both the groups. All adverse effects occurred during the period of EAS (or sham) treatment and were relieved after the completion of EAS (or sham) treatment without sequelae.

**Table 2:** Data of the adverse effects in the patients.

Adverse effects	Group EAS^a^, n=42, n (%)	Group NSAID^b^, n=41, n (%)
Patients with at least one adverse effect	22 (52)	19 (46)
Feeling hot	5 (12)	2 (5)
Pain in the sole	6 (14)	6 (15)
Arthralgia	3 (7)	2 (5)
Back pain	5 (12)	1 (2)
Limb discomfort	4 (10)	0 (0)
Muscle tightness	4 (10)	2 (5)
Musculoskeletal pain	4 (10)	1 (2)
Pain in extremity	13 (31.0)	9 (22)
Sensation of heaviness	3 (7)	1 (2)
Hypoesthesia	13 (31)	7 (17)

^a^EAS: electronic acupuncture shoes.

^b^NSAID: nonsteroidal anti-inflammatory drug.

### Primary Outcome Measurements

#### Treatment Success Rate

Treatment success was defined as the VAS score after the intervention being lower than that before the intervention. The treatment success rate in each visit is shown in Table 3. During the period from the 2nd visit to 5th visit, the NSAID and EAS groups were prescribed ibuprofen and placebo, respectively, for 7 days, and the treatment success rate in each visit was higher in the EAS group in both the ITT and PP analyses, but without significant differences. During the period from the 6th visit to 19th visit, the treatment success rate in each visit in the EAS group was higher than that in the NSAID group in the ITT and PP analyses, and significant differences were observed only during the 16th visit in the ITT analysis (EAS group: 31/37, 84% and NSAID group: 21/34, 62%; *P*=.04). The treatment success rate in the last visit (20th visit) in the EAS group was lower than that in 19th visit in both the ITT and PP analyses. The treatment success rate in the EAS group was higher than that in the NSAID group, but there were no significant differences.

**Table 3:** Data of the success rate of the treatment at each visit.

Visit	Success rate in the intention-to-treat population			Success rate in the per-protocol population		
	Group EAS^a^, n=38^b^, n (%)	Group NSAID^c^, n=34, n (%)	*P* value	Group EAS, n=33, n (%)	Group NSAID, n=30, n (%)	*P* value
2	19 (50)	16 (47)	.72	17 (52)	15 (50)	.90
3	24 (65)	21 (62)	.79	21 (64)	19 (63)	.98
4	24 (65)	21 (62)	.79	21 (64)	19 (63)	.98
5	26 (70)	19 (56)	.21	23 (70)	18 (60)	.42
6	27 (73)	22 (65)	.45	24 (73)	21 (70)	.81
7	25 (67)	22 (65)	.80	22 (67)	20 (67)	>.99
8	28 (76)	22 (65)	.31	25 (76)	20 (67)	.43
9	28 (76)	21 (62)	.21	25 (76)	19 (63)	.28
10	28 (76)	22 (65)	.31	25 (76)	20 (67)	.43
11	30 (81)	23 (68)	.19	27 (82)	21 (70)	.27
12	29 (78)	23 (68)	.31	26 (79)	21 (70)	.42
13	31 (84)	24 (71)	.18	28 (85)	22 (73)	.26
14	31 (84)	22 (65)	.07	28 (85)	20 (67)	.09
15	29 (78)	22 (65)	.20	26 (79)	20 (67)	.28
16	31 (84)	21 (62)	.04^d^	28 (85)	19 (63)	.05
17	26 (70)	20 (59)	.31	23 (70)	18 (60)	.42
18	27 (73)	21 (62)	.31	24 (73)	19 (63)	.42
19	29 (78)	20 (59)	.08	26 (79)	18 (60)	.11
20	25 (68)	20 (59)	.45	22 (67)	18 (60)	.58

^a^EAS: electronic acupuncture shoes.

^b^n=37 after the 2nd visit because the patient number 55 was excluded from the intention-to-treat analysis (the patient was not satisfied with treatment and she quitted the study).

^c^NSAID: nonsteroidal anti-inflammatory drug.

^d^Only this value was significant at *P*<.05.

#### Changes in the VAS Score From the Baseline

**Figure 3:**
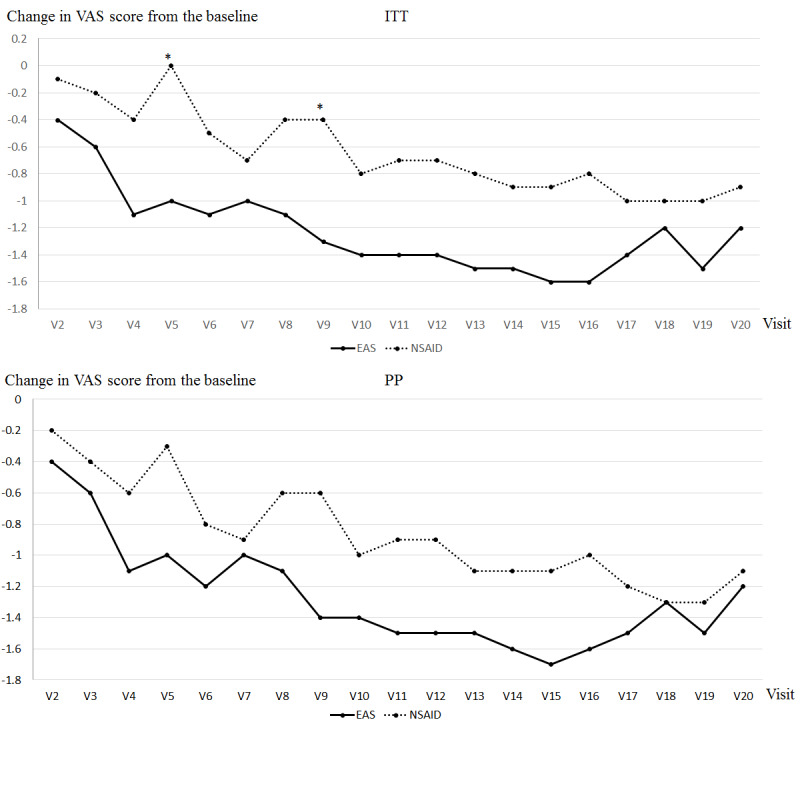
Change in visual analog scale (VAS) score from baseline in each visit in intention-to-treat and per-protocol analyses. Data are represented as means. The groups were compared at a significance level of .05. The x axis represents the day of visit for treatment, while the y axis indicates the change in VAS score from the baseline. EAS: electronic acupuncture shoes; NSAID: nonsteroidal anti-inflammatory drug; ITT: intention-to-treat; PP: per-protocol.

We calculated the change in the VAS score from the baseline after each treatment, and the results are shown in Figure 3. The VAS score at baseline was the VAS score measured during the first visit. At the first visit, mean (SD) VAS scores in the EAS and NSAID groups were 4.4 (2.46) and 4.2 (2.35), respectively, in the ITT analysis. The mean (SD) VAS scores in the EAS and NSAID groups were 4.4 (2.58) and 4.2 (2.46), respectively, in the PP analysis. No significant differences were observed between both groups. The change in the VAS score from baseline at each visit in the EAS group was greater than that in the NSAID group in the ITT and PP analyses, and significant differences were observed at the 5th visit and 9th visit in the ITT analysis (*P*=.048 and *P*=.048, respectively). In both the ITT and PP analyses, VAS scores in the EAS and NSAID groups gradually decreased during the period from the 2nd visit to the 4th visit but increased in the 5th visit. During the period from the 6th visit to 19th visit, the VAS score in the NSAID group fluctuated up and down. However, the VAS score in the EAS group continued to decrease from the 6th visit to the 17th visit and fluctuated from the 17th visit to the 19th visit in the ITT analysis. In the ITT analysis, VAS scores in the last visit in both groups were less than that at baseline. A similar trend was observed in the PP analysis.

#### VAS Scores Before and After Treatment in Each Visit

**Figure 4:**
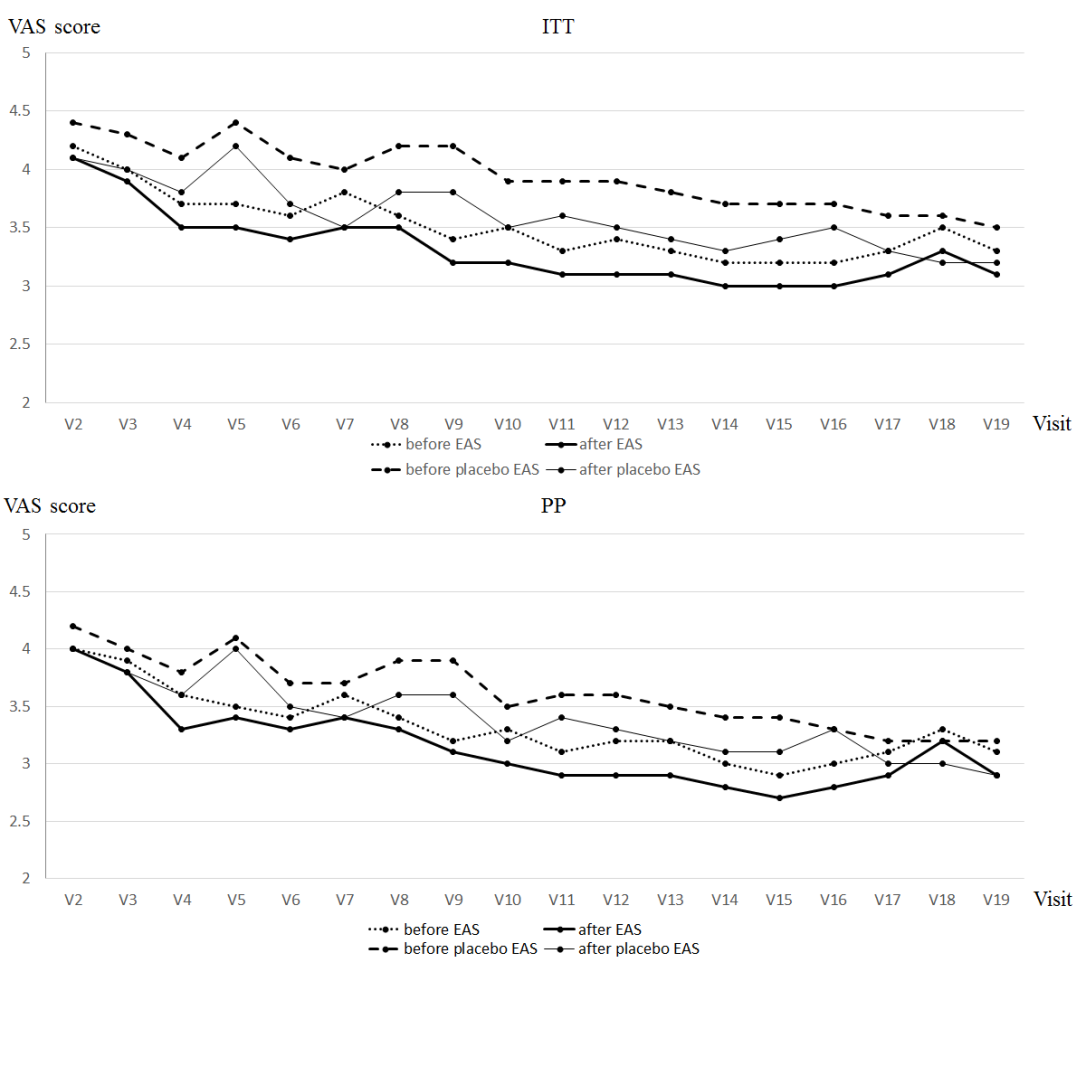
Visual analog scale (VAS) score before and after treatment in each visit in intention-to-treat and per-protocol analyses. Data are shown as means. The x axis represents the day of visit for treatment, while the y axis indicates the VAS scores. EAS: electronic acupuncture shoes; ITT: intention-to-treat; PP: per-protocol.

As presented in Figure 4, no significant differences were observed between the groups in both the ITT and PP analyses.

### Secondary Outcome Measurements

#### Time for Achieving Pain Remission

This measurement represented the course of pain remission and was defined as the time point when the VAS score began to decrease. As presented in Table 4, the time for achieving pain remission in the EAS group was 14.5 days, which was shorter than that in the NSAID group (17.4 days), but these differences were not significant in the ITT analysis. The time for achieving pain remission in the EAS group was 15.7 days, which was shorter than that in the NSAID group (16.9 days), but these differences were not significant in the PP analysis.

**Table 4:** Time for achieving pain remission.

Time for achieving pain remission	Intention-to-treat population			Per-protocol population		
	Group EAS^a^, n=38	Group NSAID^b^, n=34	*P* value	Group EAS, n=33	Group NSAID, n=30	*P* value
Mean (SD), days	14.5 (15.73)	17.4 (18.84)	.49	15.7 (16.56)	16.9 (19.07)	.79
Median days	8.0	8.0		8.0	8.0	
Range of days (min, max)	3.0, 63.0	2.0, 63.0		2.0, 63.0	2.0, 63.0	
95% CI	9.33-19.67	10.78-23.93		9.79-21.54	9.78-24.02	

^a^EAS: electronic acupuncture shoes.

^b^NSAID: nonsteroidal anti-inflammatory drug.

#### Range of Motion

**Figure 5:**
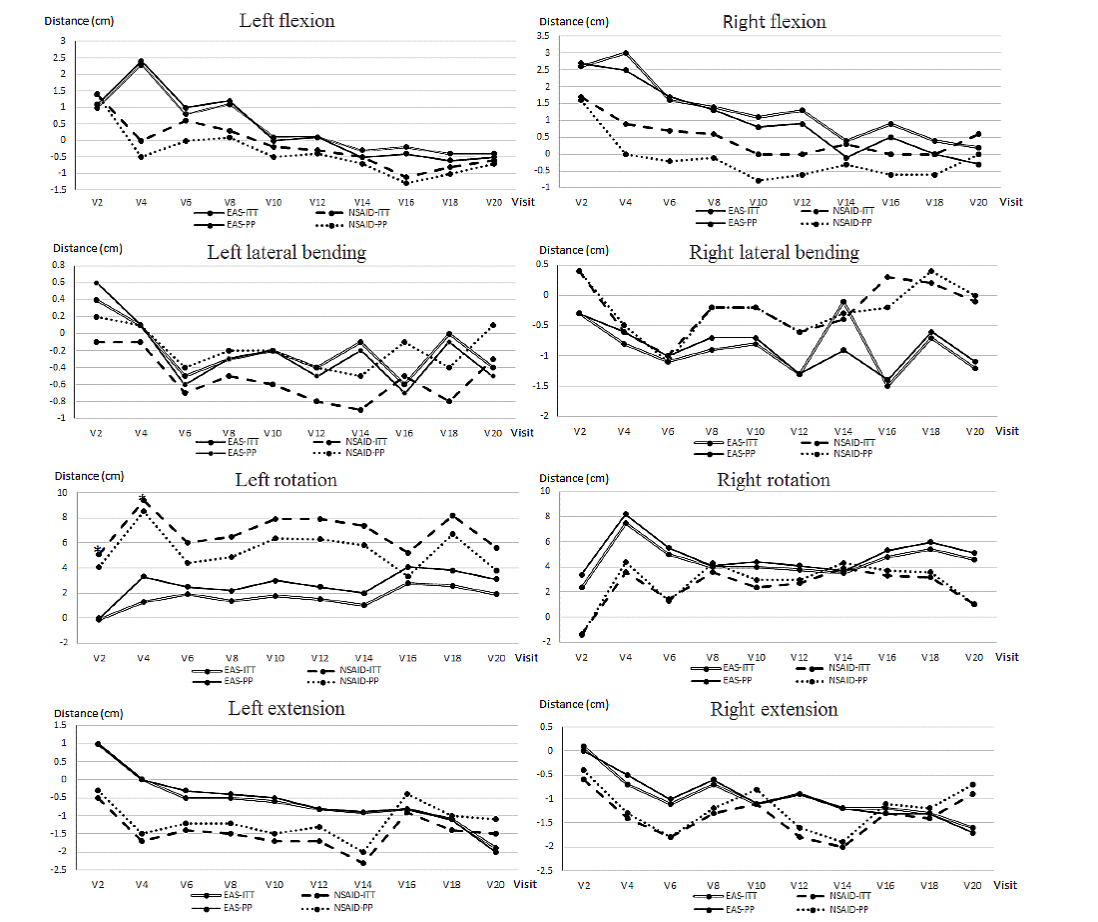
Change in range of motion from baseline in intention-to-treat and per-protocol analyses. The x axis represents the day of visit for treatment, while the y axis indicates the distance (cm). EAS: electronic acupuncture shoes; NSAID: nonsteroidal anti-inflammatory drug; ITT: intention-to-treat; PP: per-protocol.

The range of motion included right and left flexion, lateral bending, rotation, and extension. The results are shown in Figure 5. No significant differences were observed between the groups in both the ITT and PP analyses. However, significant differences were observed in the left rotation at the 2nd visit and 4th visit (*P*=.049 and *P*=.03, respectively).

#### Quality of Life and Maintaining Treatment Effect

The quality of life and maintaining treatment effect were assessed using SF-36 (Table 5) and RMDS (Table 6), respectively. All the results showed no significant differences between the groups in both the ITT and PP analyses.

**Table 5:** Quality of life assessed using the 36-item short form health survey.

Visit, SF-36^a^ scale		Intention-to-treat population			Per-protocol population		
		Group EAS^b^, n=38^c^, mean (SD)	Group NSAID^d^, n=34, mean (SD)	*P* value	Group EAS, n=33, mean (SD)	Group NSAID, n=30, mean (SD)	*P* value
**Visit 2 (baseline)**							
	Physical function	73.6 (19.96)	75.1 (16.72)	.72	75.0 (19.16)	74.8 (17.49)	.97
	Role limitation (Physical)	50.0 (43.11)	44.1 (39.91)	.55	54.5 (41.67)	44.2 (41.36)	.33
	Role limitation (Emotional)	61.4 (42.82)	56.9 (39.81)	.64	66.7 (40.82)	60.0 (39.54)	.51
	Vitality	49.1 (21.82)	50.9 (12.40)	.66	49.7 (20.32)	53.2 (10.04)	.39
	Mental health	61.4 (15.06)	63.4 (15.39)	.58	61.3 (14.34)	66.0 (14.45)	.21
	Social function	73.0 (18.50)	65.4 (21.77)	.12	72.7 (18.87)	66.7 (22.34)	.25
	Bodily pain	55.6 (19.20)	53.9 (18.29)	.70	56.5 (19.18)	55.3 (18.20)	.80
	General health	51.9 (20.55)	48.8 (18.89)	.50	53.1 (21.06)	49.6 (19.54)	.49
**Visit 14 (change from baseline)**							
	Physical function	–1.6 (11.16)	–0.3 (19.26)	.73	–1.8 (11.98)	–0.3 (20.55)	.73
	Role limitation (Physical)	1.3 (31.27)	8.1 (40.23)	.43	1.5 (33.62)	9.2 (42.79)	.43
	Role limitation (Emotional)	3.5 (36.18)	–2.0 (36.64)	.53	4.0 (38.87)	–2.2 (39.08)	.53
	Vitality	3.2 (11.07)	1.3 (11.83)	.48	3.8 (11.85)	1.5 (12.60)	.47
	Mental health	2.0 (9.05)	–0.8 (12.33)	.27	2.3 (9.70)	–0.9 (13.15)	.27
	Social function	2.3 (12.28)	4.4 (15.35)	.52	2.7 (13.17)	5.0 (16.28)	.53
	Bodily pain	3.1 (12.8)	6.8 (15.61)	.27	3.6 (13.75)	7.8 (16.43)	.28
	General health	2.4 (12.39)	3.2 (15.19)	.82	2.8 (13.28)	3.6 (16.16)	.83
**Visit 20 (change from baseline)**							
	Physical function	0.0 (12.63)	–3.2 (13.25)	.29	0 (13.58)	3.7 (14.08)	.30
	Role limitation (Physical)	3.3 (30.85)	11.0 (37.03)	.34	3.8 (33.14)	12.5 (39.25)	.34
	Role limitation (Emotional)	0 (39.52)	4.9 (37.72)	.59	0 (42.49)	5.6 (40.19)	.60
	Vitality	3.2 (15.33)	5.0 (14.09)	.62	3.8 (16.49)	5.7 (14.90)	.63
	Mental health	1.5 (8.74)	1.0 (10.15)	.82	1.7 (9.38)	1.1 (10.82)	.82
	Social function	4.3 (11.36)	0.7 (19.69)	.36	4.9(12.08)	0.8 (21.00)	.36
	Bodily pain	8.4 (15.13)	10.1 (14.71)	.63	9.6 (15.87)	11.4 (15.18)	.65
	General health	2.4 (11.84)	3.5 (14.80)	.74	2.8 (12.69)	3.9 (15.73)	.75

^a^SF-36: 36-item of a short form health survey.

^b^EAS: electronic acupuncture shoes.

^c^n=37 after the 2nd visit because the patient number 55 was excluded from the intention-to-treat analysis (the patient was not satisfied with treatment and she quitted the study).

^d^NSAID: nonsteroidal anti-inflammatory drug.

**Table 6:** Quality of life assessed using the Roland Morris Disability Scale.

Visit, Roland Morris Disability Scale		Intention-to-treat population			Per-protocol population		
		Group EAS^a^, n=38^b^	Group NSAID^c^, n=34	*P* value	Group EAS, n=33	Group NSAID, n=30	*P* value
**Visit 2 (baseline)**				.77			.57
	Mean (SD)	7.3 (4.64)	7.6 (4.62)		7.1 (4.91)	7.8 (4.88)	
	Median	6.5	7.0		6.0	7.5	
	Range (min, max)	0.0, 17.0	1.0, 18.0		0.0, 17.0	1.0, 18.0	
**Visit 14 (baseline)**				.77			.57
	Mean (SD)	7.3 (4.64)	7.6 (4.62)		7.1 (4.91)	7.8 (4.88)	
	Median	6.5	7.0		6.0	7.5	
	Range (min, max)	0.0, 17.0	1.0, 18.0		0.0, 17.0	1.0, 18.0	
**Visit 20 (baseline)**				.78			.49
	Mean (SD)	–2.1 (4.79)	–2.4 (4.62)		–1.1 (4.34)	–1.9 (4.50)	
	Median	–2.0	–1.0		0.0	–1.0	
	Range (min, max)	–11.0, 9.0	–16.0, 3.0		–11.0, 9.0	–16.0, 3.0	

^a^EAS: electronic acupuncture shoes.

^b^n=37 after the 2nd visit because the patient number 55 was excluded from the intention-to-treat analysis (the patient was not satisfied with treatment and she quitted the study).

^c^NSAID: nonsteroidal anti-inflammatory drug.

## Discussion

### Principal Results

We conducted a trial to compare the effects of EAS and conventional treatment (NSAIDs) for CLBP. In the ITT and PP analyses, the change in the VAS score from baseline in the EAS group in each visit was greater than that in the NSAID group, and significant differences were observed in the 5th visit and 9th visit in the ITT analysis (*P*=.048 and *P*=.048, respectively). In the ITT and PP analyses, the treatment success rate in each visit in the EAS group was higher than that in the NSAID group, and significant differences were observed only in the 16th visit (*P*=.04). In both the ITT and PP analyses, there were no significant differences in the VAS score before and after each treatment between the groups. No significant differences were observed in the time required for achieving pain remission, range of motion, SF-36 scores, and RMDS scores between the groups, but significant differences were observed in the left rotation at the 2nd and 4th visits.

### Comparison With Prior Work

EAS are a novel medical device, and it combine the properties of electroacupuncture and TENS and are different from acupoint TENS [8-10]. Electrodes of TENS are placed on the painful area of the body, that is, close to the lesion or near the nerve bundles proximal to the painful area [8]. Instead of local treatment, EAS are worn on the feet with electrical stimulation over 2 acupoints (KI1 and *shimian*) on the soles. Moreover, the administration of both conventional TENS and acupuncture-like TENS is required to achieve physiological intentions (paresthesia and muscle twitch, respectively) to confirm its effectiveness, thereby making patient blinding impossible [8-10]. By contrast, EAS outputs complex waveforms composed of low-frequency and middle-frequency waves. Furthermore, the amplitude of the current is so low that patients wearing EAS barely notice they are under treatment.

In clinical practice, needling on *yongquan* (KI1) is usually employed for treating conditions of disturbance of consciousness [20]. Few studies have evaluated the effect of electroacupuncture on KI1 [21]. *Shimian* is an extra acupoint, not located on the meridian, and it is employed to treat insomnia. According to the theory of traditional Chinese medicine, the flow of defensive *qi* (*wei qi*) travels around the outer side of the body (*yang*) through a special pattern and streams into the inner side of the body (*yin*) through the kidney meridian [22-24]. Moreover, in traditional Chinese medicine, one mechanism underlying insomnia is that the defensive *qi* cannot stream from the *yang* to *yin* [22,23]. Thus, many acupoints of the kidney meridian are believed to regulate the flow of defensive *qi* and are used to treat insomnia [25,26]. According to the theory of traditional Chinese medicine, chronic pain is a result of disharmony or depletion in the supply of *qi* [27,28]. For these reasons, we suspected that the regulation of the circulating defensive *qi* might relieve chronic pain. Therefore, we chose the *yongquan* and *shimian* as our treatment acupoints and applied electric current to these areas.

Yang et al [12] conducted an animal study, wherein the results revealed that the application of TENS on KI1 could produce analgesic effects; this was demonstrated through the increased response time of hind paw licking to thermal stimuli induced by complete Freund’s adjuvant. The results of immunostaining and western blotting of the brain and spinal tissue of rats revealed that the application of TENS on KI1 inhibited ERK2 activation and c-Fos expression, which are associated with pain perception. In that study, TENS was not applied as a conventional method to the painful lesion or nearby nerve bundles but on an acupoint distal to the painful site. Stimuli were transferred through the peripheral afferent fiber to the central nervous system and they caused changes in the brain network, thereby affecting pain perception and modulation [13]. These findings can explain that EAS may alleviate CLBP through a central pathway.

Furthermore, we think that EAS also work possibly through a peripheral pathway. Application of acupuncture on acupoints remote from the lesion or site of pain to alleviate disease or uncomfortableness is a fundamental concept based on the meridian theory in traditional Chinese medicine. A study demonstrated that acupuncture on distal acupoints could increase the blood flow of the meridian distribution area that the acupoints belong to [14]. A few studies found that CLBP is correlated with insufficient blood flow in the lumbopelvic region [15,16]. To summarize these theories and findings, we speculate EAS may alleviate CLBP by improving the blood flow.

The strengths of our study are as follows. First, we designed a randomized, double-blinded, placebo-controlled, dual-intervention (real EAS with placebo analgesic and sham EAS with ibuprofen) crossover trial to investigate the effect of EAS compared with that of conventional NSAIDs. Achieving participant blinding is very difficult in the design of an electroacupuncture or TENS study unless patients have little knowledge of electroacupuncture or TENS [8]. In this study, all patients were unaware of EAS because EAS are innovative medical devices. Even if patients were aware of EAS, they still could not differentiate between the real and sham treatment because the applied current was too small to be felt. Second, we employed rigorous methodology in sample size calculation and analytical paradigms (ITT and PP) to evaluate the effects of the intervention [29]. Third, we obtained various outcome measurements to compare the effects of EAS and NSAIDs; we focused not only on the pain itself but also on the mental status and the range of motion affecting the quality of life of the patients.

### Limitations and Future Directions

Our study has several limitations. First, we did not test the success of blinding by using an assessment tool such as that developed by Deyo et al [30], although patients were not knowledgeable about EAS and the applied current was extremely low. Second, the duration of ibuprofen intake was only 1 week in order to prevent the side effects such as gastrointestinal, cardiovascular, renal, hepatic, and other systemic adverse effects [31-35]; therefore, we could not determine whether a longer intake of ibuprofen would achieve a higher efficacy. In clinical practice, the duration of NSAID use in patients with CLBP should be examined and evaluated regularly [36]. Treatments for CLBP include pharmacological, interventional, and surgical strategies [36]. Oral NSAIDs are widely used as first-line therapy for CLBP [36]. However, for specific populations with CLBP, the risk of adverse effects and drug-drug interactions [37-41] should be considered before prescribing oral NSAIDs. In this situation, developing an alternative therapy with similar efficacy to NSAIDs is crucial. The effect of TENS on CLBP is still debatable [42]; although the effectiveness of acupuncture is highly recommended [43], it depends on the performer.

EAS might serve as a reliable alternative therapeutic tool. Additional studies for evaluating the effect of EAS on other sites or categories of pain such as neck pain or pain caused by cancer should be conducted. EAS are more than just a tool for treating LBP. If sensors can be integrated to monitor physiological parameters and then shared on the internet, physicians will be able to personalize the amount of electrical stimulation through remote control [44]. This system will make a great contribution to mobile health, and in this era of new infectious diseases such as COVID-19, this feature will enhance the applicability of EAS [45].

### Conclusion

In this prospective double-blinded randomized controlled study, we demonstrated that EAS had a better treatment success rate and analgesic effect compared to NSAIDs in patients with CLBP during some of their visits for treatment, with partial improvement in the range of motion. EAS could be considered as a potential noninferior and reliable alternative therapeutic tool for patients with CLBP who are contraindicated for oral NSAIDs.

### Data Availability

There are restrictions on the availability of data for this study owing to the signed consent agreements regarding data security, which only allow access to external researchers for research monitoring purposes. Requestors wishing to access the trial data to replicate or check our analyses can apply to Protech Pharmaservices Corporation (contact@ppccro.com) after receiving permission from the East Bamboo Company Limited (ingo@eastbamboo.com.tw) and principal investigator Doctor Yu-Sheng Chen (cusp01@cgmh.org.tw).

## Appendix

Multimedia Appendix 1 CONSORT checklist.

## Abbreviations

CLBP: chronic low back pain
CONSORT: Consolidated Standards of Reporting Trials
EAS: electronic acupuncture shoes
ITT: intention-to-treat
LBP: low back pain
NSAIDs: nonsteroidal anti-inflammatory drugs
PP: per-protocol
RMDS: Roland Morris Disability Scale
SF-36: 36-item short form
TENS: transcutaneous electrical nerve stimulation
VAS: visual analog scale
